# Answer to Dr. Drake’s Interrogatories

**Published:** 1853-07

**Authors:** 


*Answer to Dr. Drake's Interrogatories.—Continued.*

The local causes of decay, to which we have alluded, exist more or less in every section of our country, and in every land. Man is nowhere exempt from disease, and wherever disease exists, the dental organs, with the rest making up the general system, suffer. That they are more frequently subject to decay in the west, than in other sections of the United States, would, perhaps, be difficult to prove. If locality in this country has any effect in inducing decay, we should expect that place to be more sickly, and especially as it regards that class of diseases which prove so destructive in childhood. There are, of course, localities prejudicial to general health, and we think the teeth suffer proportionably.

From the local causes named, it must be apparent that cleanliness would have much to do in preventing decay. The teeth must be very imperfectly organized, indeed, and the secretions of the mouth in a very vitiated condition, if they exhibit much disposition to decay on those points, where corroding agents cannot remain on them. We are led to believe that if they could be kept as clean in their indentations, and where they press together, that we should find them decayed but seldom. This view is confirmed by the fact, that those who are most careful in keeping them clean at these points, preserve their teeth best; and it is known that the Brahmans, who are reputed to have the best teeth in the world, make it a part of their religious worship, to spend sometime every day in cleaning their teeth, and in this, they use a twig of soft wood, which effectually removes from between the teeth and elsewhere, all extraneous matter; the enamel is thus kept polished by mechanical means, with a substance not hard enough to wear it away.

We have not particularly alluded to all the external or local causes of decay; enough has, however, been said to determine the class of agents of this kind, which would seriously injure these organs. We have alluded to the locality on the teeth, where we expect most frequently to meet with disease, and named indentations and approximal surfaces as these points; from this would be inferred that a crowded condition of the teeth is a predisposing cause of decay. The question here arises: Does the pressure, induced by a crowded condition of the teeth, break up the regular crystalized enameled prisms, and in this way make it more liable at this point to be acted upon by corroding agents? If so, this must take place before the eruption of the teeth, because the crown is formed; before this, and after the protrusion through the gum, no deposition of enamel can take place. The pressure, however, may not be great until after the teeth make their appearance through the gums—but is not the enamel too hard after this to yield to the amount of force occasioned by a crowded condition of the teeth? We think it is; and hence, attribute the decay here taking place, to the retention of corroding substances.

In a crowded condition of the teeth, decay generally manifests itself in early life, and most frequently at that period of life when the individual feels but little the importance of good teeth, and cannot well appreciate the advantage to be derived from a rigid system of cleanliness. It does not take a continued state of pressure and negligence for years to produce injurious effects. If the teeth are so perfectly organized as to resist these causes for several years, we may not expect that they will in after life be injured so readily by it. Teeth imperfectly organized and very much crowded in the mouth, should be watched with a great deal of care, and often examined by the dentist, for one not accustomed to make such examinations will not be able to detect incipient decay—for it is not always dark, and, indeed, the enamel may be decomposing without any discoloration which would attract attention.

Under the second general question you also ask:

"Is Dyspepsia a cause of early decay?" and "Does the acid thrown up by many dyspeptics, in paroxisms of that disease, act chemically on the teeth?"

These interrogatories come under the same general head, and can be answered together. The mere fact, which we believe observation fully confirms,—that those who suffer much from this disease, have generally bad teeth—leads us to inquire into the cause, and this leads to the inquiry, What is the condition of the secretions of the mouth? Are they generally, of an acid character, and is the acid to which you refer "thrown up in paroxisms of this disease," of a character to act chemically on the teeth?

The gastric juice has been the subject of much experiment, and from the fact that it is generally conceded to be the solvent for food taken into the stomach, its importance cannot well be over-estimated. In its normal state, Schwann, from a series of experiments, was led to believe that the gastric juice contained a "peculiar substance," which he called *pepsin*, and which, co-operating with an acid, "becomes the solvent fluid essential in the process of digestion. Prout, as the result of his analysis, shows the free acid of the gastric juice to be the muriatic. Gruelin and Tiedeman have found it to contain the acetic, and Simon the lactic; and Simon remarks, "that from the researches of the latter chemist, which are the most perfect we possess on the subject, it appears that in addition to the free acids, the gastric juice contains mucus, and occasionally (in horses) a very small quantity of albumen, extractive and salivary matter, and that the ash consists of alkaline, muriates and sulphates, a little phosphate and sulphate of lime, chloride of calcium, magnesia and peroxide of iron." He further remarks, that the "gastric juice collected from the empty stomach, although mixed with mucus, was tolerably clear; it was neutral, of a yellow color, a saline taste, and on evaporation left only 23 of solid constituents. Gastric juice obtained by irritating the stomach with pebbles was acid, viscid, and of a pale-brown color. Hiinefeld does not believe that there is any free hydrochloric acid in gastric juice." Dunglison has, however, not only detected this acid in the gastric juice, but also, the acetic and phosphoric in combination with potash, soda, lime, and magnesia.

From the somewhat varied results exhibited from the investigation of our best chemist, we cannot but regard the presence of the different alkalies, with the kind of diet taken into the stomach, as exerting some influence in presence of the free acid found in this fluid. If, for instance, two or more acids were found in the stomach at the same time, and lime or soda was introduced, that having the greatest affinity to these, will unite with them and leave the other in a free state. This condition of things might be changed by the presence of other substances.

Mr. Blondlot "believes that 'the digestive property of gastric juice depends, not on its obvious chemical constitution, but on a peculiar organic principle. If exposed to a temperature of 104° to 120° F., or higher, it loses entirely and irrevocably its digestive power, although to all appearance, and even so to its composition, as made known by analysis, it remains unchanged." And further, "that the action of the stomach in coagulating milk, is not due to its digestive principle solely, but to its acid, which acts like lactic acid. That the effect of the gastric juice upon bones, whether entire or not, is to disintegrate them slowly, beginning at the surface, and to reduce the earthy matter into a fine chalky powder, but without dissolving or decomposing it.*

It appears that this fluid, when concentrated by evaporation, becomes strongly acid, effervesing with chalk, and not losing its acid reaction when an excess of this is added.

From the repeated experiments which have been made with this fluid, there can be no doubt that it contains an acid or acids, and they are all of that class which, when brought in contact with the teeth, will act upon them with more or or less rapidity. What effect then would be produced on this fluid when in a morbid condition? Is it more acid or alkaline? Simon remarks, "that the only modifications respecting, which we can speak with any degree of certainty, are the following: 1st, There may be a considerable excess of free

*See Simon's Chemistry of Man, page 317.

acid; 2d, there may be a diminution of free acid; and 3d, the gastric juice may be positively alkaline. In all probability, with these are associated other changes in the composition of the fluid, producing an injurious effect on the process of digestion; but on this subject we are unable to speak with certainty." And he goes on further to remark, that "the increased acidity of the gastric juice usually arises from excess of those acids which exist in its normal state, namely, muriatic, acetic and lactic. When there is a tendency to the formation of an excess of acid in the gastric juice, it appears to be developed from the food. Muriatic acid is principally developed from animal food; acetic and lactic from vegetable, and especially saccharine food, such as acid bread, beer, and wine, and the fatty acids from an excessive use of fatty matters. An excessive acidity of the gastric juice is frequently observed in cases of gastritis serosa, and of scrofula and rickets associated with disease of the spleen. In gout, podagra, and nettlerash, the gastric juice contains, according to Stark, phosphoric and uric acids; the presence of the latter acid must, however, be regarded as very problematical.

"The cases in which the gastric juice exhibits a positively alkaline reaction, are comparatively rare. This deviation from the normal condition, arises chiefly from the use of salted or putrid food and drink, containing basic salts, from prolonged fastings, and especially from care and anxiety." (Stark.)

We may safely infer that in a large majority of cases where gastric irritation exists, that we have an increased acidity of this fluid, and when this condition occurs, and the peculiar form of the disease is such as to frequently bring this in contact with the teeth, injury must result therefrom.

In those cases of gastric dyspepsia, where the disease consists principally in disordered functions of the stomach, and where heartburn and acid eructations are a troublesome concomitant of the disease, the secretions which are continually about the teeth, become vitiated, and it would require more than ordinary care to keep the teeth from injury. Local remedies exert in these cases only a secondary influence, and nothing short of general treatment, correcting the diseased condition of the stomach, can be expected to prove permanently beneficial, not but that dentrifices and washes, if properly compounded, may not be in such cases of some service; yet, their application being only occasional, their effect cannot be more than temporary.

Prout, in treating of the development or presence of acids in the stomach,* remarks that "the acids found in the stomach are derived from two sources—from the blood circulating in the vessels supplying the stomach, or from changes occurring in the matters secreted by these vessels, and from the alimentary matters taken into that organ. The lactic and muriatic acids are principally derived from the blood and from the matters secreted or introduced into the stomach, while the oxalic, butyric, acetic, carbonic, and perhaps, in some cases, the lactic acids, are developed from the food during its imperfect assimilation; which imperfect assimilation is often a concomitant circumstance attending the abnormal development of the lactic and muriatic acids. Hence, in cases of severe dyspepsia, accompanied by great acidity, the acids present have, possibly, in all cases, a double origin." The same author thinks that the muriatic and lactic acids are always present in the stomach, during the reducing process of assimilation.

He thinks also, that the predominance of the muriatic acid in the stomach denotes a phlogestic or inflammatory state of the system, while a predominence of the lactic denote a state of irritation. And that in dyspeptics of a plethoric habit and gouty disposition, the muriatic acid generally predominates This subject, as connected with the assimilation of our food, and the pathology of this particular class of diseases which so directly acts upon the secretions of the mucous membrane and salivary organs, is of particular interest and great importance to the Dentist.

*See Prout on the Stomach and Renal Diseases, page, 81

Bad teeth are generally by the Dental profession regarded as one of the most frequent causes of dyspepsia. This, we have no doubt, is generally owing to the fact that dyspeptics so often have bad teeth and from the continued cause operating to induce decay, their teeth are so very difficult to preserve. We have no doubt there is a reflex influence, both from disease of the stomach on the teeth, and vice versa. The teeth are indeed the organs of prehension for the alimentary system, receiving at first their germination from the mucous membrane which is alike distributed throughout the alimentary canal, observation and reason both confirm the fact that disease, affecting this to any great extent, and for any considerable length of time, exerts also an influence on these organs. The sympathy which exists between the gums and stomach, is forcibly shown in the painful dentition of children. Here the entire nervous system suffers, and the mucous membrane takes on general disease often resulting in death.

As illustrative of the effect of dental irritation on Dyspepsia, I present a case as given by Dr. Chapman, in his work on the thoracic and abdominal viscera. He remarks, "that many years ago I was consulted by a lady from Natchez for the disease, (Dyspepsia,) of long continuance, and of a very harrassing nature. Discovering that her gums were highly irritated, from a very bad state of her teeth, I suspected them to be the cause of the suffering, and had all the carious ones extracted, by which, without any medicine, she in a short time recovered. But in a few months, having an artificial set, which being badly adjusted, was productive of much irritation, the dyspepsia returned with equal violence, and again ceased on the removal of the teeth. These being subsequently well arranged, she has since worn them without any recurrence of the disease."

Such cases as the above are indeed not so very rare. We have met with numerous cases where dyspepsia has been relieved and often entirely cured, by a judicious and thorough dental operation. We think it is important that a Dentist should know if his patient is dyspeptic, that he may be enabled to adopt a proper course of treatment, for the preservation of the teeth, and equally important that the physician should know the condition of the teeth that he may adopt such treatment for the cure of his dyspeptic patient, as the pathological conditions of the system indicate.

In taking the view of decay of the dental organs we have presented, any change in the normal condition of those fluids which are constantly brought in contact with the teeth, rendering them more acid, must be pernicous. In dyspepsia, the mucous membrane—even if the disease is primarily nervous as some contend, necessarily suffers and all the secretions thrown upon its surface by the glandular system, or thrown off more directly by the membrane itself, become vitiated, and as before remarked, tend to keep up diseased action. In all those cases therefore of Dyspepsia, where the secretions are rendered more acid, it is a cause of decay, and this embraces a large majority of the complicated forms of this disease, and particularly in the acute stage thereof. In some of the chronic conditions of this disease, when the vital energy has been much exhausted, the secretions are supposed not to be so strongly acid, some times even becoming alkaline; the same injury would not then result to the dental organs. Before this sets in as a sequence to an aggravated form of acute disease, there is often found not many teeth to be injured. Bad teeth in many ways must tend to impair digestion. First, by the irritation to the mucous membrane, extending to the stomach, and causing an excess of acid, the salvia necessarily swallowed also being of an acid character; the imperfect comminution of the food, etc., etc.

